# Robot-assisted endoscopic removal of a huge tricuspid valve myxoma: case report

**DOI:** 10.1186/s13019-022-01978-5

**Published:** 2022-10-06

**Authors:** Lun-Wu Hung, Cheng-Ya Lee, Hiong-Ping Hii, Nan-Chun Wu, Bor-Chih Cheng

**Affiliations:** 1 Division of Cardiovascular Surgery, Department of Surgery, Chi Mei Medical Center, No. 901, Zhonghua Rd., Yongkang Dist, 710 Tainan City, Taiwan; 2 Division of Cardiovascular Surgery, Department of Surgery, Tungs’ Taichung MetroHarbor Hospital, No.699, Section 8, Taiwan Boulevard, Wuqi District, Taichung City, 43503 Taiwan

**Keywords:** Morcellator, Myxoma, Minimally invasive, Robotic, Case report

## Abstract

**Background:**

Cardiac myxoma is the most common benign cardiac tumor. Its tremendous size and fragile character severely bother the surgeons. Several minimal invasive approaches had been applied for radical tumor excision. The wound was forcibly enlarged for en-bloc specimen removal and prevention of debris sputtering.

**Case presentation:**

We reported a case of huge tricuspid valve (TV) myxoma managed by robot-assisted endoscopic tumor resection and TV repair, with initial presentation of worsening shortness of breath for two months. The tumor was downsized with a morcellator and removed through a keyhole wound (1.1 cm in diameter). The patient recovered uneventfully and was discharged after four days.

**Conclusions:**

With the first morcellator application, this might be the smallest surgical wound reported after the removal of a huge cardiac myxoma. The ICU and hospital stays were shortened. This might be effectively applied to further minimally invasive surgeries for cardiac tumor excision.

**Supplementary Information:**

The online version contains supplementary material available at 10.1186/s13019-022-01978-5.

## Background

Myxoma is the most common benign cardiac tumor. The incidence was reported to be 60–80% at the left atrium, 15–28% at the right atrium, 8% at the right ventricle, and 3–4% at the left ventricle [1]. The atrioventricular valve area was the least common origin. There are several approaches to remove myxoma, including traditional sternotomy, minimally invasive thoracotomy, and robot-assisted surgery. The emerging approaches are for achieving smaller operation wounds, less wound pain, shorter ICU and hospital stay, and better cosmetic outcomes. By introducing the morcellator (NOUVAG AG, Inc, Goldach, Switzerland), we’d made endoscopic huge tumor removal possible and more accessible.

## Case presentation

**Fig. 1:**
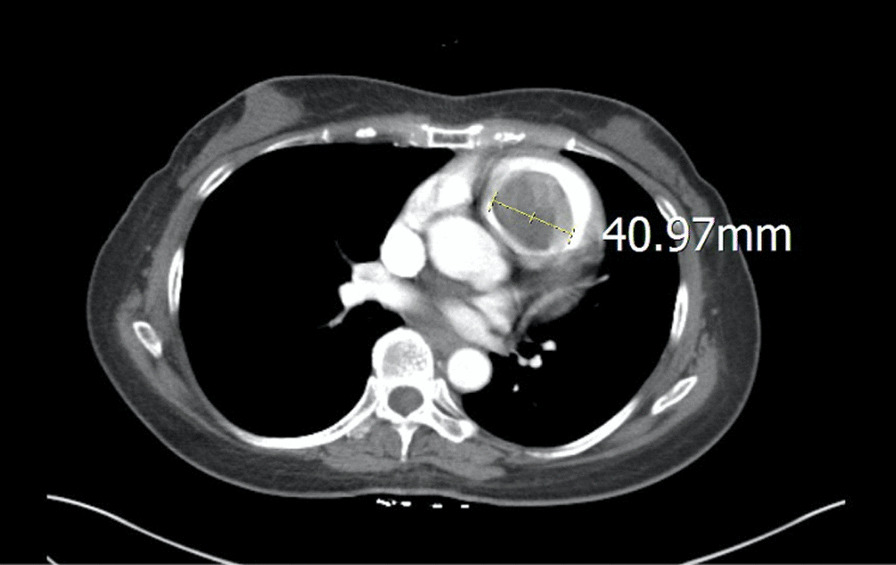
CT scan showing a huge myxoma originating from the tricuspid valve and extending into the right ventricular outflow tract and pulmonary trunk, with a maximum diameter of about 41 mm

A 56 year-old woman, without known systemic disease, visited our out-patient department for worsening shortness of breath for two months. A grade II/VI systolic murmur was noted over the 2nd left parasternal border. Jugular vein engorgement was also noted. The patient was a government employee and she had no habit of smoking, alcohol or drug abuse. There was also no family history of cardiac tumors. Transthoracic echocardiogram showed a huge mobile tumor in the right ventricle, originating from the TV and extending into the pulmonary trunk. The tumor stalk, about 12 mm in diameter, was connected to the anterior tricuspid leaflet and its chordae tendineae. Myxoma was suspected. The tumor occupied the right ventricular outflow tract and nearly obstructed the pulmonary valve. The estimated tumor volume based on computed tomography (CT) scan was 69.36 cm^3^, with a maximum diameter of 41 mm (Fig. 1). An urgent operation was conducted for unstable hemodynamic status.

The operation was performed using a totally endoscopic, robot-assisted approach. After the induction of general anesthesia, the patient was intubated smoothly. A central venous catheter and 6.5 Fr introducer sheath were placed into the right internal jugular vein. A right groin incision was made for peripheral cannulation. After systemic heparinization, the patient’s femoral artery was cannulated (19 Fr) for systemic retrograde perfusion. Adjunctive distal femoral perfusion was done using an 8 Fr arterial cannula (Medtronic, Minneapolis, MN). Bicaval venous drainage was initiated through the right internal jugular vein (17 Fr) and femoral vein (23 Fr). Cardiopulmonary bypass was established. A camera port (7 mm) was introduced into the fourth intercostal space (ICS) at the right midclavicular line. The left and right robot arm ports (7 mm) were placed in the second ICS at the midclavicular line and sixth ICS at the anterior axillary line. The assisting port (7 mm) was placed in the fourth ICS at the right parasternal border. The Da Vinci Xi surgical system (Intuitive Surgical, Inc, Sunnyvale, CA) cart was docked. A 11.5 mm endoscopic trocar port (Thoracoport™, Medtronic, Minneapolis, MN) was placed at the right fourth ICS. A transthoracic Chitwood cross-clamp (Scanlan International, Minneapolis, MN) was placed at the right fourth ICS at the mid-axillary line. A cardioplegia delivery pig-tail catheter was inserted through the second ICS at the right parasternal border directly into the ascending aorta. Right pericardiotomy was done. After aortic cross-clamping, myocardial protection was achieved using antegrade cold crystalloid cardioplegia with histidine-tryptophan-ketoglutarate solution (30 cc/kg; Custodiol HTK; Köhler Chemie GmbH, Bensheim, Germany). The heart was decompressed by bi-caval snare. Right atriotomy was done. Atrial retractor was applied, and the TV was exposed.

**Fig. 2:**
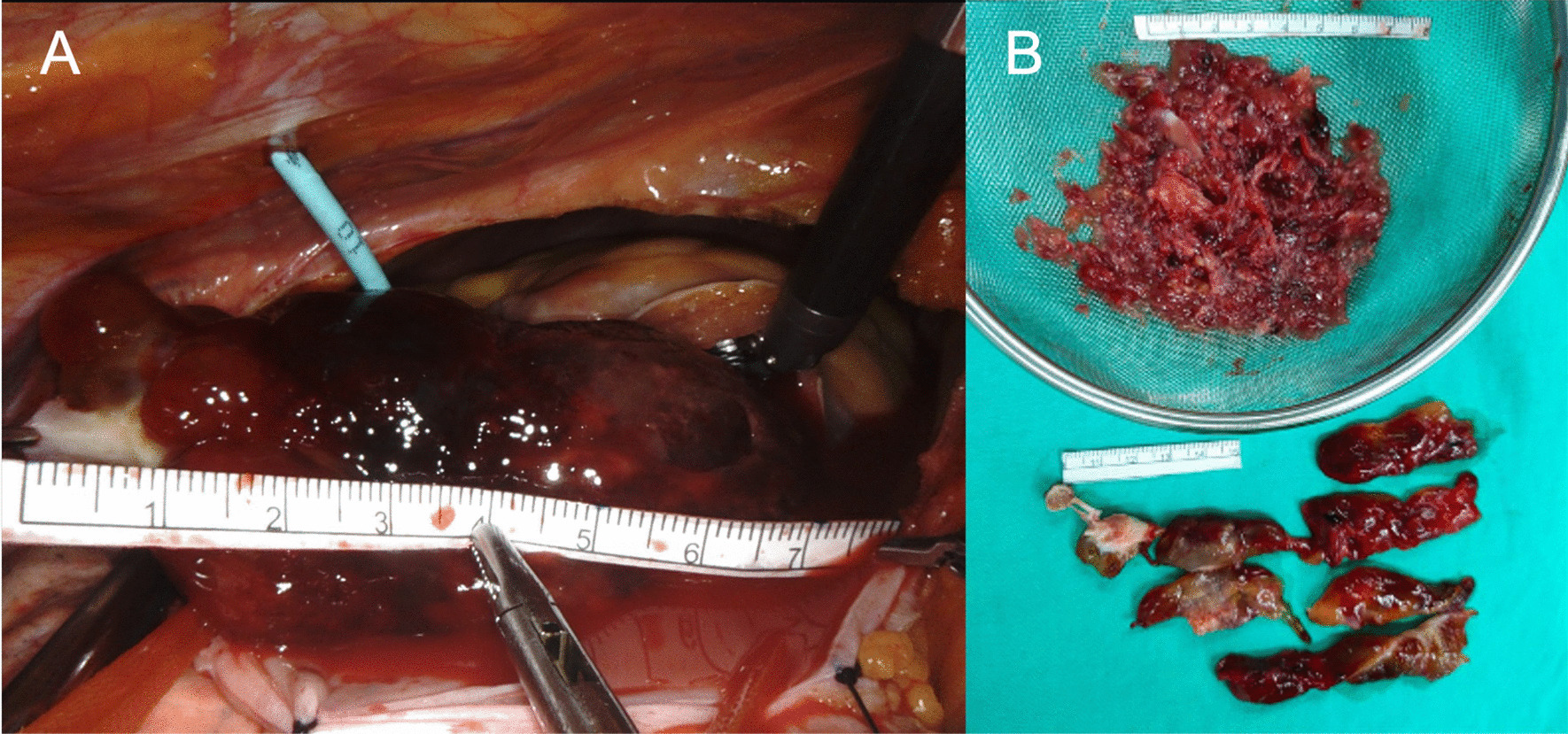
**A** The myxoma, about 8 cm in length, was retracted into the pericardial cavity. **B** The tumor specimen removed by a morcellator via the trocar working port

When exploring the tumor, the anterior leaflet of the TV was incised perpendicularly in the midline. The tumor stalk, about 12 mm in diameter, connects the ventricular side of the anterior leaflet and associated papillary muscle. Hence, the tumor was excised en-bloc with the associated leaflet and papillary muscle tip. It was placed in a wired endo bag [UNIMAX, Taipei, Taiwan (R.O.C.)] inserted from the working port. Then, the tumor was cut into pieces by the morcellator and sucked out without debris sputtering (Fig. 2) (Additional file 1).

**Fig. 3:**
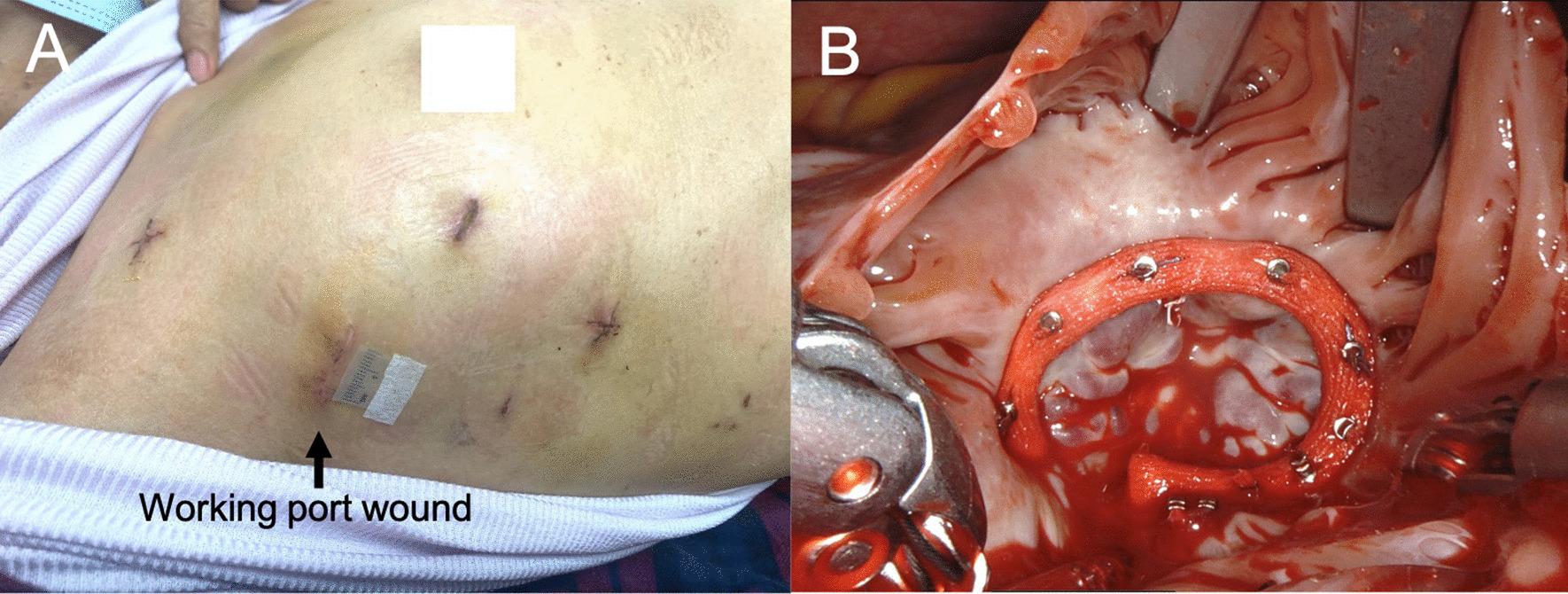
**A** This postoperative photo shows the standard minimal thoracotomies of the Da Vinci system. The marked working port for tumor retrieval is 1.7 cm in length after suturing, with an original diameter of about 1.1 cm. **B** Tricuspid valve repair with artificial cords and valvuloplasty ring

The anterior leaflet was repaired with CV-5 sutures. One CV-5 artificial cord was applied between the residual papillary muscle and corresponding leaflet edge. A 28 mm annuloplasty ring (MC3, Edwards Lifescience, Irvine, CA) was applied with 10 sets of Cor-knots (LSI SOLUTIONS, Victor, NY) fixation (Fig. 3). Normal sinus rhythm resumed after aortic clamp off. The patient weaned off bypass without inotropes. Transesophageal echocardiogram showed no tricuspid regurgitation after heart rhythm resumed. The aortic clamping time was 121 min, and the total cardiopulmonary bypass time was 175 min.

Extubation was done two hours after the surgery. The patient was transferred to the general ward on the next day and was discharged uneventfully on the fourth postoperative day.

The pathology report confirmed the diagnosis of cardiac myxoma. During one-year follow up period, there was no residual symptom. The transthoracic echocardiogram one year after the operation showed no residual tricuspid regurgitation or tumor recurrence.

## Discussion and conclusions

TV tumor is a rare disease. In a collective review, only 51 cases of myxoma originating from the TV were reported by 2008. Most tumors that originated from the TV required TV repair or replacement and myxoma resection (replacement, 22%; repair, 56.1%; tumor resection only, 22%) [2, 3]. The tumor size varies from 1 to 15 cm in diameter. The larger tumor also appears to be a major risk factor for embolism [4].

In the past two decades, minimally invasive techniques were applied in all areas of cardiac surgery due to admirable cosmetics and speedy postoperative recovery [5]. The Da Vinci robot-assisted cardiac surgery provides stable scope, enhanced visualization by ten-fold, and dexterous surgical arms. Nevertheless, the chest wounds must be widened in extra-large tumor excision despite the minimal operations designed.

The morcellator was initially approved for laparoscopic uterine myoma removal. However, the device was less used recently as per FDA recommendation due to potential malignant tissue spreading. The FDA recommends performing laparoscopic power morcellation for myomectomy only within a well-protected containment system.

In this case, the huge myxoma was excised en-bloc and placed into the endo bag within the pericardial cavity. A morcellator shaft (12 mm in diameter) was inserted into the endo bag with an attached negative pressure filter system. The downsized tumor strips were removed smoothly with confined debris. In only 5 min, the procedure was completed through the tiny working port.

To our knowledge, there was no previous report on morcellator-assisted cardiac tumor downsizing and port removal. With the first morcellator application, this might be the smallest surgical wound reported after the removal of a huge cardiac myxoma. With the Da Vinci system, the ICU and hospital stays were shortened. The most concerned cosmetic problems and wound pain were also alleviated. Removal of tumor with calcification or tense fibrosis parts are limitations that need further investigation. Overall, this approach provides an effective option to further minimally invasive surgeries for cardiac tumor excision.

## Supplementary Information

**Additional file 1.** Illustrated the comprehensive steps of the surgery. Perioperative transesophageal echocardiography finding, tumor exploration, tumor morcellation, and the final tricuspid valve repair were all dipicted.

## Data Availability

Not applicable.
